# Disseminated *Scedosporium boydii* infection with pulmonary and cerebral involvement in a patient with COPD: A case report

**DOI:** 10.1016/j.mmcr.2025.100737

**Published:** 2025-09-20

**Authors:** Parviz Hassanpour, Seyed Jamal Hashemi, Zahra Ramezanalipour, Sanam Nami, Behrooz Naghili

**Affiliations:** a Department of Parasitology and Mycology, School of Public Health, Tehran University of Medical Sciences, Tehran, Iran; b Food Microbiology Research Center, Tehran University of Medical Sciences, Tehran, Iran; c Department of Parasitology and Mycology, Faculty of Medicine, Tabriz University of Medical Sciences, Tabriz, Iran; d Infectious and Tropical Diseases Research Center, Tabriz University of Medical Sciences, Tabriz, Iran

**Keywords:** *Scedosporium boydii*, Antifungal susceptibility, Obstructive pulmonary disease

## Abstract

A 51-year-old Iranian man survived a severe respiratory illness but later died from a brain abscess caused by *Scedosporium boydii*, identified post-mortem. The case highlights the importance of comprehensive care, including adherence to treatment, regular follow-ups, and the use of both culture-based and molecular diagnostics. Incomplete treatment likely contributed to the fatal outcome. A key limitation of this report is the absence of MRI images due to a technical malfunction, preventing further radiological assessment. This case stresses the need for sustained and thorough medical management in complex infections to improve patient outcomes.

## 1 Introduction

*Scedosporium boydii* (*S. boydii*) is a filamentous fungus that commonly causes a broad spectrum of clinical diseases, particularly in immunocompromised hosts. Common sites of infection include the lungs, skin, sinuses, eyes, bones, joints, and central nervous system (CNS). When *S. boydii* invades the CNS, both diagnosis and treatment become exceptionally challenging due to its resistance to many antifungal agents, the limited availability of effective therapeutic options, and the generally poor prognosis. Early identification and appropriate treatment can reduce the mortality rates. Here, we present a case of brain abscess (BA) in a chronic obstructive pulmonary disease (COPD) patient caused by *S. boydii*. Historically, *Candida* species and *Cryptococcus neoformans* were the most commonly identified fungal pathogens in CNS infections [1,2]. However, in immunosuppressed patients, especially those with underlying diseases, various opportunistic fungi have recently emerged as significant pathogens. Among these, *S. boydii* is a relatively rare but well-documented CNS pathogen. *Scedosporium boydii* (previously known as *Allescheria boydii* and *Petriellidium boydii*) and its asexual form, *S*. *boydii*, are ubiquitous saprophytic fungi commonly found in soil, manure, decaying vegetation, and polluted water sources [3,4]. When *S. boydii* infection involves the CNS, it most commonly presents as multiple or solitary parenchymal BA. Despite the prompt diagnosis and aggressive therapeutic intervention, brain abscesses caused by *S. boydii* demonstrate an overwhelmingly fatal prognosis. This case represents a rare documentation of a BA caused by *S. boydii* in a patient with COPD from the Northwest of Iran, contributing valuable insights to the limited clinical literature on this challenging infectious condition.

## 2 Case presentation

The present study is based on a case report of a selected patient from among 360 individuals with underlying pulmonary diseases who were hospitalized in various medical centers in Northwest Iran between 2023 and 2024 [5]. This case report focuses on a patient who developed a brain abscess. The study received approval from the Ethics Committee of Tehran University of Medical Sciences (Ethics Committee Protocol Number: IR.TUMS.SPH.REC.1402.106). Written informed consent was obtained from the patient's family.

### 2.1 Case

A 51-year-old male patient, with a history of COVID-19 infection resulting in COPD, was admitted to a tertiary care hospital in northwestern Iran for comprehensive medical evaluation and management. He was an ex-smoker with a history of hypertension, but remained physically active. His medical history included the use of hydrochlorothiazide (12.5 mg daily) for hypertension, warfarin for chronic atrial fibrillation, and home oxygen therapy at a rate of 2 L/min. On day 0 (Day 1 of hospitalization), He was admitted to the pulmonary disease center with severe symptoms of shortness of breath, cough with bloody sputum, and fatigue. Laboratory findings at the time of admission showed mild leukocytosis of 14,500/mm^3^ with a predominance of neutrophils (80 %). Chest computed tomography (CT) revealed mucus obstruction and bronchiectasis. On admission, the patient was started on empirical antibiotic therapy with ceftriaxone (1 g intravenously once daily) and azithromycin (500 mg intravenously once daily) for two days. There were no signs of improvement. On day 2, a bronchoalveolar lavage (BAL) specimen was collected by a pulmonologist using standard bronchoscopy and sent to the laboratory. Examination under the microscope with 10 % KOH revealed dichotomous branching at 45°, indicating a fungal and invasive agent, but on day 4, culture on Sabouraud Dextrose Agar (SDA) medium only grew *Candida* spp. and *Streptococcus* spp. Also, galactomannan was tested on serum using ELISA (*Platelia™ Aspergillus Ag, Bio-Rad, France*). The optical density index (ODI) value was <0.5, which is considered negative according to the manufacturer's guidelines. Given the microscopic findings and clinical context, a diagnosis of probable invasive pulmonary fungal disease was considered despite negative fungal cultures. Given the presence of septate dichotomous branching hyphae observed in the BAL sample and clinical suspicion of invasive pulmonary aspergillosis, antifungal therapy with intravenous voriconazole (VRC) was initiated at a loading dose of 6 mg/kg every 12 hours for two doses, followed by a maintenance dose of 4 mg/kg every 12 hours. Simultaneously, antibacterial therapy with ceftriaxone was continued for appropriate coverage of *Streptococcus* spp. After 12 days (On day 14) and achieving partial clinical improvement, antifungal therapy with oral VRC (200 mg twice daily) was continued for a total treatment duration of 6–12 weeks. The patient declined further hospitalization despite medical advice and was discharged against medical advice (AMA) with incomplete antifungal therapy. The potential risks of premature discontinuation of treatment, including relapse or progression of invasive pulmonary aspergillosis, were thoroughly explained to the patient, who provided informed consent.

**Fig. 1:**
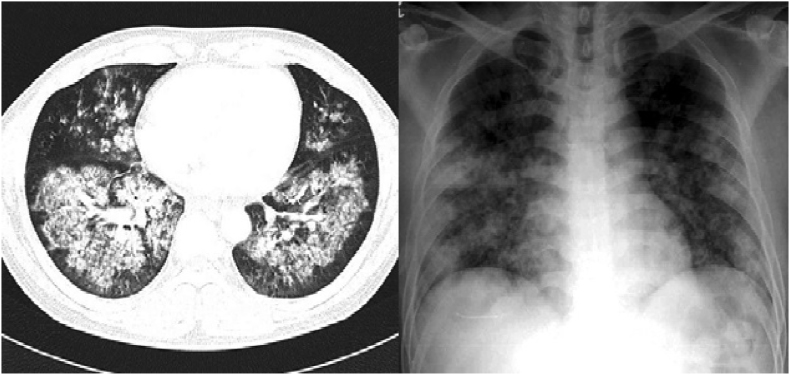
Chest radiography and CT confirmed the pre-existing chronic obstructive pulmonary disease. Chest CT showed parenchymal infiltrates through both lung fields on admission. Chest radiography showed patchy consolidation infiltration in both lung fields on admission.

**Fig. 2:**
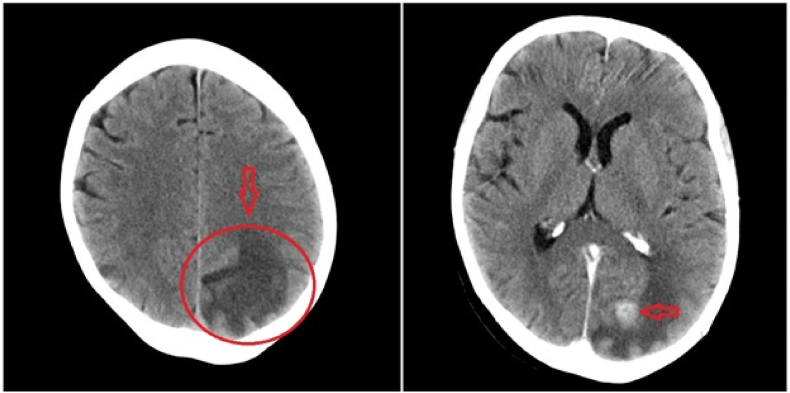
A brain CT scan, non-contrast multiple areas of decreased attenuation in the bilateral occipital lobes and the right parietal lobe (red arrows).

On day 28, two weeks after discharge against medical advice, the patient presented to a referral center in northwestern Iran with high-grade fever above 38 °C and severe unilateral headache. Given the clinical suspicion of otitis media, the patient was admitted to the intensive care unit (ICU) for close monitoring and management. The patient's prior history of invasive disease and incomplete antifungal therapy raised concerns for possible complications or secondary infections. The patient was conscious, and physical examination revealed a stiff neck and a positive Kernig's sign. The initial diagnosis was chronic suppurative otitis media with meningitis. The patient also had unilateral otalgia, purulent ear discharge, and decreased hearing, which were suggestive of chronic suppurative otitis media. On day 29, chest radiography and CT confirmed the pre-existing chronic obstructive pulmonary disease (Fig. 1). Routine blood parameters, including erythrocyte sedimentation rate (ESR), C-reactive protein (C-RP), and white blood cell (WBC) count, were abnormal. In a brain CT scan, a solitary ring-enhancing lesion, approximately 15–20 mm in diameter, was identified in the left occipital lobe on neuroimaging. Surrounding vasogenic edema was prominent and involved both the occipital and parietal lobes on the left side (Fig. 2). Due to the unavailability of MRI imaging at the facility, the patient was evaluated and followed up based solely on brain CT findings in conjunction with laboratory results and clinical presentation. On day 30, cerebrospinal fluid (CSF) (3 tubes, 2 ml per tube) was transported to the laboratory in an unrefrigerated biohazard bag. CSF results, showing elevated protein levels, normal glucose levels, and sterile cultures. The protein in the CSF of the patient was 0.84 g/l (normal range, 0.12–0.60 g/l), glucose 3.2 mmol/l (normal range, 2.2–3.9 mmol/l), and no growth in SDA culture. This finding led to a diagnosis of chronic suppurative otitis media complicated by a BA. Considering the patient's history of invasive pulmonary fungal infections and laboratory findings, a fungal brain abscess was suspected. Therefore, treatment was initiated with intravenous voriconazole (VRC; 6 mg/kg every 12 hours as a loading dose, followed by 4 mg/kg every 12 hours for maintenance), along with intravenous ceftriaxone 2 g every 12 hours for antibacterial coverage. Given the lesion size and stable neurological status, surgical intervention was deferred pending response to medical therapy. Close radiological and clinical monitoring was planned. However, on day 34, due to clinical deterioration and lack of improvement despite appropriate medical therapy, surgical intervention was performed for abscess drainage. The aspirate and secretions were sent to the laboratory for microbiological and histopathological examination. Due to disease progression and increased intracranial pressure, the patient underwent surgery, was admitted to the ICU, placed on mechanical ventilation, and, unfortunately, passed away one day later (day 40).

### 2.2 Microbiological examination and molecular identification

**Fig. 3:**
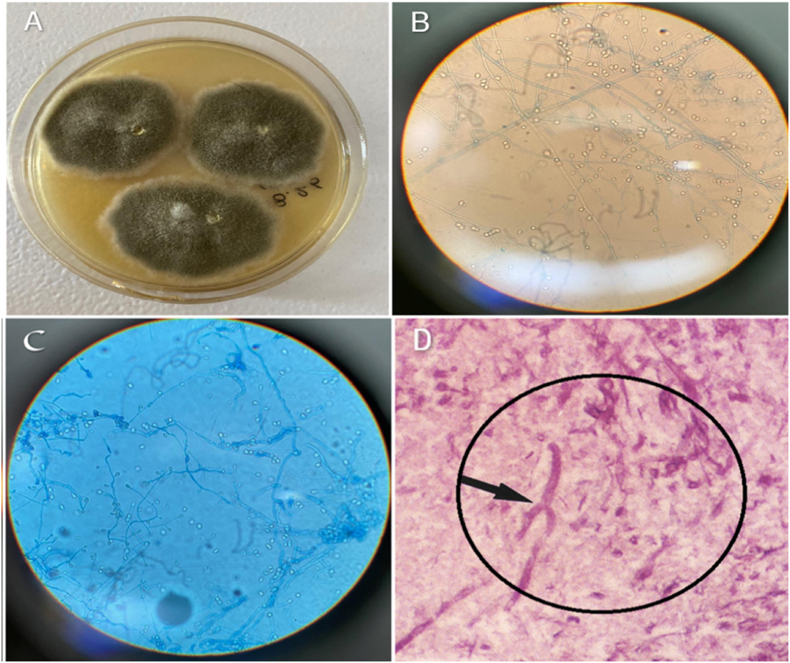
Microscopic appearance, culture, and histopathological analysis results of the abscess Sample. (A) White to tan, cottony colonies on SDA after 6 days of incubation. (B) Conidiophore of *Scedosporium boydii* with a solitary oval to pyriform conidium (KOH preparation). (C) Acute-angle branching septate hyaline hyphae with lateral conidiation (lactophenol cotton blue preparation). (D) Septate, branching hyphae with granulomatous inflammation (H&E staining).

Hematoxylin and eosin (H&E) staining, Gram stain, bacterial culture, smear, and fungal culture were performed on the specimen (Fig. 3). Histopathological examination revealed septate and branching hyphae accompanied by granulomatous inflammation. Microbiological examination revealed fungal elements upon microscopic analysis with KOH 10 %. Cultures on the SDA medium showed a grayish, velvety colony with short hyphae and a reverse grayish-black coloration. Further examination using lactophenol cotton blue revealed numerous unicellular, pale brown conidia, both singly and in small groups, located on elongated, simple, and branched conidiophores or laterally on hyphae, consistent with *S. boydii*. The DNA was isolated from the mycelium of pure culture colonies using the phenol-chloroform technique [5]. The organism was confirmed as *S. boydii* through a comprehensive molecular diagnostic approach. Specifically, panfungal PCR targeted the internal transcribed spacer (ITS) region of the ribosomal DNA gene sequence, followed by definitive DNA sequence analysis for precise species confirmation. The sequences were aligned using the Basic Local Alignment Search Tool (BLAST) and compared against fungal sequences available in GenBank. database to assess their similarities. The generated ITS sequence was ultimately deposited in GenBank under accession number PV845593 (*S. boydii*).

### 2.3 In-vitro antifungal susceptibility testing (AFST)

AFSTs of VRC (*Sigma-Aldrich*, USA), Itraconazole (ITR) (*Sigma-Aldrich*, USA), caspofungin (CAS) (*Sigma-Aldrich*, USA), and Amphotericin B (AMB) (*Sigma-Aldrich*, USA) were conducted according to the Clinical and Laboratory Standards Institute (CLSI) guidelines (CLSI M38-A2) [6]. The Minimum inhibitory concentrations (MICs) were as follows: VRC (0.03 μg/ml), CAS (0.12 μg/ml), ITR (0.5 μg/ml), and AMB (8 μg/ml). Breakpoints have not been defined for this pathogen; however, in vitro susceptibility suggested VRC and CAS as potentially active agentsin this study.

## 3 Discussion

Fungal infections of the CNS are uncommon but are associated with high mortality, mainly due to delayed diagnosis, limited therapeutic options, and resistance of causative fungi [1,7]. Among these, *S*. *boydii* has emerged as an important opportunistic pathogen capable of causing disseminated disease involving the lungs and CNS. Although most reported cases occur in immunocompromised hosts, such as those with organ transplants, hematological malignancies, or diabetes, infections in immunocompetent individuals have also been documented, particularly following near-drowning incidents or traumatic events [[8], [9], [10]].

Our case highlights COPD as the most likely predisposing factor, with initial pulmonary involvement progressing to CNS dissemination. Similar to previous reports, the clinical presentation was nonspecific, including fever, headache, and neurological signs, which complicates early diagnosis [11]. Radiological imaging identified a ring-enhancing brain lesion; however, due to a technical malfunction, MRI imaging was not available, and the diagnosis relied on CT scans, surgical observations, and pathological data, which together provided sufficient information for clinical decision-making. In another case report, a *S. apiospermum* brain abscess was documented in a 69-year-old immunocompromised male with a history of silicosis [12]. Silica exposure impairs alveolar macrophage function, which is critical for early recognition and immune response to inhaled fungal conidia, thereby preventing parenchymal invasion. By analogy, it is plausible that our patient, who had COPD, became colonized as a result of impaired macrophage function, which facilitated the dissemination of the fungus from the respiratory system to the CNS.

Definitive diagnosis of CNS scedosporiasis remains challenging. In our patient, histopathology and culture demonstrated typical fungal structures, and sequencing of the ITS region confirmed *S. boydii*. Historically, *S. boydii* and *S. apiospermum* were considered synonyms, but they are now recognized as distinct species, underscoring the importance of precise molecular identification. This highlights the value of integrating microscopy, culture, and molecular tools in routine diagnostic workflows [13,14]. Voriconazole is generally considered the first-line antifungal due to its favorable CNS penetration and in vitro activity [13]. Our isolate showed susceptibility to VRC and CAS; however, breakpoints for *Scedosporium* spp. are not well established. Despite appropriate antifungal therapy, the patient's outcome was poor, largely due to incomplete treatment during the first admission, which likely facilitated relapse and dissemination. Similar unfavorable outcomes have been described in recent reports despite aggressive treatment, highlighting the critical role of strict adherence to therapy and prolonged follow-up [9,10]. Although chronic suppurative otitis media is frequently associated with *Pseudomonas* spp., in our patient the available pre-treatment microbiological data (BAL culture) indicated *Streptococcus* spp., and no *Pseudomonas* spp. were isolated from respiratory or CSF samples. Therefore, ceftriaxone was selected for its activity against streptococci and favorable CNS penetration. We acknowledge the absence of a pre-treatment middle-ear swab as a limitation that could have further guided empiric antibacterial selection. In conclusion, this case illustrates the diagnostic and therapeutic complexity of CNS scedosporiasis and emphasizes the importance of early recognition, multidisciplinary diagnostic approaches, and adherence to long-term antifungal therapy. Strengthening clinical awareness and improving laboratory capacity are essential to reduce the high mortality associated with this emerging fungal infection.

## CRediT authorship contribution statement

**Parviz Hassanpour:** Writing – original draft, Validation, Investigation, Formal analysis, Data curation. **Seyed Jamal Hashemi:** Supervision. **Zahra Ramezanalipour:** Writing – review & editing, Software, Investigation. **Sanam Nami:** Validation, Conceptualization. **Behrooz Naghili:** Supervision.

## Consent

Written informed consent was obtained from the patients for publication of this case series and accompanying images. A copy of the written consent is available for review by the Editor-in-Chief of this journal on request.

## Ethical statment

This study was financially supported by the Tehran University of Medical Sciences, (Grant No: IR.TUMS. SPH.REC.1402.106).

## Funding

This study was financially supported by the Tehran University of Medical Sciences (Grant No: IR.TUMS.SPH.REC.1402.106).

## Declaration of competing interest

The authors declare no conflicts of interest or personal relationships that could have appeared to influence the work reported in this paper.
